# Evidence for a Developmental Role for TLR4 in Learning and Memory

**DOI:** 10.1371/journal.pone.0047522

**Published:** 2012-10-11

**Authors:** Eitan Okun, Boaz Barak, Ravit Saada-Madar, Sarah M. Rothman, Kathleen J. Griffioen, Nicholas Roberts, Kamilah Castro, Mohamed R. Mughal, Mario A. Pita, Alexis M. Stranahan, Thiruma V. Arumugam, Mark P. Mattson

**Affiliations:** 1 The Mina and Everard Goodman Faculty of Life Sciences, The Leslie and Susan Gonda Multidisciplinary Brain Research Center, Bar-Ilan University, Ramat Gan, Israel; 2 Department of Neurobiology, Life Sciences Faculty and the Sagol School of Neuroscience, Tel Aviv University, Tel Aviv, Israel; 3 Laboratory of Neurosciences, National Institute on Aging Intramural Research Program, Baltimore, Maryland, United States of America; 4 Department of Physiology, Georgia Health Sciences University, Augusta, Georgia, United States of America; 5 School of Biomedical Sciences, The University of Queensland, Queensland, Australia; 6 Department of Neuroscience, Johns Hopkins University School of Medicine, Baltimore, Maryland, United States of America; Centre d'Immunologie de Marseille-Luminy, CNRS-Inserm, France

## Abstract

Toll-like receptors (TLRs) play essential roles in innate immunity and increasing evidence indicates that these receptors are expressed in neurons, astrocytes and microglia in the brain where they mediate responses to infection, stress and injury. Very little is known about the roles of TLRs in cognition. To test the hypothesis that TLR4 has a role in hippocampus-dependent spatial learning and memory, we used mice deficient for TLR4 and mice receiving chronic TLR4 antagonist infusion to the lateral ventricles in the brain. We found that developmental TLR4 deficiency enhances spatial reference memory acquisition and memory retention, impairs contextual fear-learning and enhances motor functions, traits that were correlated with CREB up-regulation in the hippocampus. TLR4 antagonist infusion into the cerebral ventricles of adult mice did not affect cognitive behavior, but instead affected anxiety responses. Our findings indicate a developmental role for TLR4 in shaping spatial reference memory, and fear learning and memory. Moreover, we show that central TLR4 inhibition using a TLR4 antagonist has no discernible physiological role in regulating spatial and contextual hippocampus-dependent cognitive behavior.

## Introduction

Toll like receptors (TLRs) are type-I trans-membrane receptors that are best known as sensors of microbe-associated molecular patterns (MAMPs) by cells of the innate immune system [1]. In addition, TLRs recognize damage-associated molecular patterns (DAMPs), also termed ‘endogenous ligands’, generated in response to traumatic tissue injury or as a by-product of inflammation [2]. Binding of MAMPs or DAMPs to TLRs typically activates signaling cascades that result in production of inflammatory cytokines/chemokines by effector cells, and may also stimulate a peripheral immune response [3]. TLR4, a widely studied TLR, is activated by bacterial lipopolysaccharide (LPS), a constituent of the outer membrane of gram-negative bacteria. Central activation of TLR4 by LPS has been thoroughly studied and was shown to reduce hippocampal pyramidal neuron dendrite length and to impair hippocampal-dependent spatial reference memory in an inflammation-dependent manner implying a neuroinflammatory role for TLR4 following activation with bacterial-derived ligands [4], [5].

Increasing evidence indicates that TLRs located in the central nervous system are involved in developmental and adult neuroplasticity even in the absence of activation by infectious agents or tissue damage [6]. TLR3 for example, is a negative regulator of embryonic neural progenitor cell (NPC) proliferation [7]. TLR2 and TLR4 are expressed in adult NPCs [8] and have distinct and opposing functions in NPC proliferation and differentiation; TLR2 deficiency impairs hippocampal neurogenesis, whereas TLR4 deficiency enhances proliferation and neuronal differentiation [8]. Further, TLRs 2, 3 and 4 are expressed in hippocampal neurons [9], and we showed recently that TLR3-deficient mice exhibit enhanced hippocampus-dependent working (but not reference) memory, coupled with extended retention of spatial reference memory [10]. In contrast, TLR3-deficient mice demonstrate impaired amygdala- and anxiety-related behavior [10]. Because TLR3 is involved in neurogenesis, NPC proliferation and cognitive learning and memory, we sought to determine the involvement of TLR4 in these processes.

The hippocampus, which possesses a well-defined neuroanatomy, is involved in various types of learning, which are governed by different strategies [11], [12]. While TLR4 is involved in CNS plasticity processes such as NPC proliferation, the impact of TLR4s on various aspects of hippocampus-dependent learning and memory remains unclear. Here we provide evidence that TLR4 is involved in modulating hippocampus-dependent learning and memory. By measuring performance of TLR4-deficient (TLR4^−/−^) and wild-type (TLR4^+/+^) mice in tests of hippocampus-dependent cognitive function, we provide evidence that developmental TLR4 deficiency enhances spatial reference memory but impairs contextual fear conditioning. In contrast, antagonism of TLR4 in adult TLR4^+/+^ mice has no impact on cognition, and instead affects anxiety responses.

## Methods

### Animals

Young adult male congenic TLR4^−/−^ mice (B6.B10ScN-*Tlr4^lps–del^*/JthJ) (*n* = 19) and their respective wildtype mice (C57bl/6) (*n* = 24) were purchased from Jackson Laboratories (Bar Harbor, ME, USA). The TLR4^−/−^ mice we used were backcrossed to C57bl/6 mice for 6 generations in the original laboratory where they were generated, as well as backcrossed an additional 7th generation in the Jackson Laboratory, followed by 7 inbreeding generations (see: http://jaxmice.jax.org/strain/007227.html). All experiments were completed using mice at 2–4 months of age. Animal care and experimental procedures followed NIH guidelines and were approved by the National Institute on Aging Animal Care and Use Committee.

### Behavioral testing

#### Spatial learning

To evaluate spatial learning and memory, mice were tested in variants of the Morris Water Maze (MWM) paradigm [10]. In all spatial learning tests, mice were first tested for their performance in a visible platform variant to exclude the possibility that the tested mice are impaired in visual ability or motivation, and to facilitate their habituation to the water pool. Mice were trained in the a water-filled pool (160 cm diameter) for 3 consecutive days, 4 trials per day, without spatial cues on the walls of the room, but with a black metal object attached to a visible platform (153 cm2 area), which was located above water-level. Mice were allowed to swim freely for 60 s, and mice unable to find the visible platform were placed on the visible platform for 10 seconds. To test for spatial reference memory, mice were trained in the pool for 5 consecutive days, 4 trials per day, with spatial cues on the walls of the room. The cues (see [10] for details) were black and white only, to reduce possible effects of color discrimination capabilities between the different genotypes. The platform was hidden 0.5 cm below the surface of the water; the water was made opaque using nontoxic white paint to prevent the mice from seeing the platform. The water was maintained at a temperature of 27±0.5°C, and constant temperature was ensured by mixing the water every 1.5 hours, and supplementing warm water when needed. The platform location was constant and starting points were changed every trial to avoid track memorization. When trials ended, either when the mouse had found the platform or when 60 seconds passed, mice were allowed to rest on the platform for 30 seconds. After completion of the 5-day learning phase, mice were tested for memory retention of the platform location. The platform was removed and mice were allowed to swim freely for 60 seconds. These ‘probe trials’ were conducted 24 and 48 hours after the last trial and every day afterwards until memory of the platform location was extinct in all experimental groups, which was indicated by equal mean distance from the platform when compared between groups. Data on mean distance from the platform as well as all other criteria was provided by the Anymaze software.

In the visible platform, reference and working memory variants of the MWM, tests were conducted under conditions of 20 lux to reduce stress to the mice. In experiment involving cannulations, the cannulated mice were allowed 7 days to recover from the surgery before testing in the MWM. In all the MWM tests, mice that exhibited passivity or thigmotaxic swimming pattern were excluded from analysis [10].

#### Open Field Test

Open field arena testing was performed using the MEDOFA-MS system (Med Associates, St Albans, VT, USA). Animals were placed in the center of an open field (40.64 cm ×40.64 cm) and exploration was assessed for 15 min. Cages were cleaned with ethanol following each session. The peripheral 10.16 cm of the zone were considered as the peripheral zone and the central 20.32 cm^2^ were considered as the central zone. All open field tests were conducted under light intensity of 400 1ux.

#### Rotarod test

Rotarod tests were performed using the ENV-577M system (Med-associates, St. Albans, VT, USA), in a brightly lit room (1300 lux). Rotarod acceleration was set to 4–40 revolutions per minute (RPM). Mice were placed on the rotarod for three 5-minute trials with 15 minutes rest between trials, and time until the first fall was recorded. The apparatus was routinely cleaned with water and ethanol following each session.

#### Fear Conditioning

Before testing, mice were first habituated to the testing room for 3 h/day for 3 days. In the training session, mice were placed in a contextual conditioning chamber (model MEDVFC-NIR-M; Med Associates) placed inside sound attenuating boxes (model MED). The conditioning chambers contained a metal grid on the floor (context A) and mice were allowed to explore the chamber for 2 min. At the end of 2 min mice were subjected to three sessions of audio tone (CS, conditioned stimulus) and foot shock (US, unconditioned stimulus). Audio tone (5 kHz, 70 dB) was on for 30 s, followed immediately by a 0.5-mA, 2-s foot shock from the metal grid floor. Thirty seconds separated each session. Foot shock intensity was determined in a preliminary test on a separate cohort of animals for the minimal applicable intensity to achieve a minimal freezing threshold of 40% during contextual fear. On the following day, in the contextual fear session (context A), mice were returned to the conditioning chamber for 5 min without any shock or audio tone. The percentage of time freezing was recorded and used as an index of *contextual memory*. In the cued conditioning (conducted 3 h following contextual conditioning) mice were returned to the chamber but in a different context (context B). Context B was comprised of plastic triangle inserted to the cage coupled with plastic flooring placed underneath the triangular plastic insert. Mice were allowed to explore the chamber for 5 min without any audio tone. Following this, five audio tones were played for 30 s each. The percentage of time freezing until and after the audio tones was recorded and used as an index of cued memory. In all fear conditioning tests, cages were cleaned with ethanol between tests.

#### Elevated Plus Maze

The apparatus consisted of a plus-sign shaped maze elevated 60 cm from the floor and comprised of two opposite open arms, 25 cm ×5 cm each, crossed by two arms of the same dimensions enclosed by 30-cm-high walls. In addition, a 1-cm-high edge made of clear Plexiglas surrounded the open arms to avoid falls. Each mouse was placed in the middle of the maze facing the open arm. Following 5 min of testing, mice were returned to their home cages. Arm preference was automatically analyzed using the Anymaze video tracking software (Stoelting, USA), and time spent in each arm and number of arm entrances were recorded. Elevated plus maze experiments were conducted under a light intensity of 1,300 lux to promote anxiety in the mice and apparatus was cleaned with ethanol between tests.

#### Intracerebroventricular cannulation and TLR4 antagonist infusion

A chronic indwelling cannula was implanted into the lateral ventricles of mice to allow intracerebroventricular (ICV) application of TLR4 antagonist (inhibitory LPS from R. Sphaeroides, Invivogen, San Diego, CA, USA) or artificial cerebrospinal fluid (aCSF). When applying this antagonist at concentrations 10–100 fold higher than LPS, effective competition over the binding site on MD-2 is achieved. We chose a concentration of 1 μg/μl because it was previously shown by Rodgers KM [13] and colleagues that an even lower concentration of 0.2 μg/μl successfully antagonizes LPS in the brain in-vivo. Cannulae (brain infusion kit 3) and micro-osmotic minipumps (model 1004) were purchased from Alzet (DURECT). At 2 months of age, mice were anesthetized with isoflurane, and a cannula was implanted and fixed on the surface of the skull using dental cement. The tip of the cannula was located in the lateral ventricle (distance from the bregma: anteroposterior, −0.25 mm; lateral, 1 mm; depth, 2.5 mm, according to [14]) for ICV infusion. aCSF (*n* = 10) or TLR4 antagonist [1 μg/μL], dissolved in aCSF (*n* = 10)] was delivered using a subcutaneous micro-osmotic pump (model 1004) attached to the ICV cannula that provided a 0.1 μL/h flow rate.

#### Immunoblots

Lysates of cultured cortical neuronal cells were obtained by washing cells in ice-cold PBS and resuspending them in a modified RIPA lysis buffer (150 mM sodium chloride, 50 mM Tris-HCl pH 7.4, 1 mM EDTA, 1% Triton-X100, 1% sodium deoxicholic acid, 0.1% sodium dodecylsulfate, 5 µg/ml aprotinin, 5 μg/ml leupeptin, 1 mM phenylmethylsulfonyl fluoride). Protein concentration was determined using the Bradford reagent (BioRad, Hercules, Canada). Protein samples (50 μg) were prepared and electrophoresed on a 12% Bis-Tris gel. Proteins in the gel were then transferred to a PVDF membrane. Membranes were blocked in 5% non-fat milk for 1 h at room temperature (RT), followed by an overnight incubation at 4°C with primary antibodies against: CREB, (1∶5000, Cat # 9197, Cell signaling), phospho-CREB (1∶2000, Cat # 9198, Cell Signaling), ERK1/2 (1∶400, Cat # 9102, Cell signaling), phospho-ERK1/2 (1∶500, Cat # 9106, Cell signaling), GluR1 (1∶10000, Cat # AB1504, Millipore). Phospho-Ser945 GluR1 (1∶1000, Cat # AB5849, Millipore), in 0.05% sodium azide and 1% bovine serum albumin in TBS with 0.1% Tween-20 at RT for 1 h. Protein bands were visualized using a chemiluminescence detection kit (Amersham Biosciences, Piscataway, NJ, USA).

#### Statistical analysis

Analyses of the MWM (except probe trials), fear-conditioning and rotarod experiments were performed using two-way *ANOVA* repeated measures with a Bonferroni post-hoc test. ANOVA statistical analysis was performed using Prizm 5 (Graphpad, USA). Correlation matrices were generated for Latency to reach the hidden platform and mean distance from the platform versus swim speed. An analysis of covariance (ANCOVA) was used to determine whether the observed effects were still significant after controlling for the variance contributed by the correlated variables. Analysis of co-variance (ANCOVA) and Pearson's correlation was performed using SPSS (IBM, USA). Probe trials in the MWM test were analyzed using one-way *ANOVA* repeated measures, with a Bonferroni post-hoc test to verify that the time mice spent in the target quadrant where the platform was located was significantly longer than in all 3 other non-targeted quadrants. All other data in this study were analyzed using unpaired two-tailed Student's *t*-test. Results are expressed as mean ± S.E.M.

## Results

### TLR4 deficiency improves spatial memory acquisition

**Figure 1:**
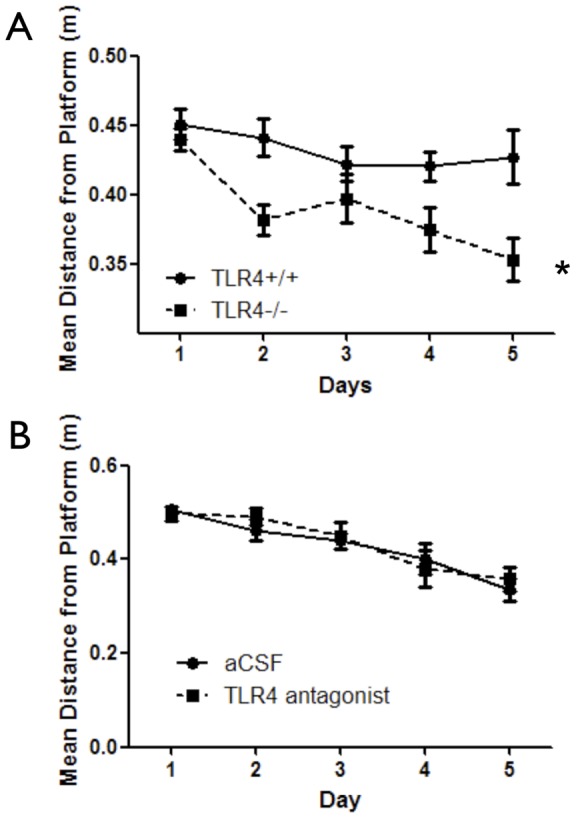
Developmental TLR4 deficiency enhances spatial reference memory. (**A**) TLR4^−/−^ (*n* = 19) and TLR4^+/+^ (*n* = 24) mice were trained in the spatial reference memory hidden platform variant of the MWM during 5 consecutive days with 4 trials per day. TLR4^−/−^ mice demonstrated improved spatial navigation capabilities compared with TLR4^+/+^ mice as indicated by significantly shorter mean distance from the platform. (**B**) Mice (C57BL/6) were implanted with an osmotic pump containing either aCSF or a TLR4 antagonist (*n* = 10 per group). The pump was connected via tubing to a cannula, which was positioned to the lateral ventricle. Mice were trained in the spatial reference memory hidden platform variant of the MWM over 5 days with 4 trials per day. No difference was measured in mean distance from the platform. *p<0.05 (Two-way RM-ANOVA).

To determine whether TLR4 affects cognitive behavior, we first compared long-term spatial memory acquisition abilities between TLR4**^−/−^** and TLR4**^+/+^** mice using a reference memory variant of the MWM task [15]. TLR4 deficiency resulted in significantly shorter mean distance from the hidden platform (F_(*1,164*)_ = 12.01, *p* = 0.0013) (Figure 1A) and shorter latency to reach the hidden platform (F_(*1,164)*_ = 7.36, *p* = 0.0097) (Figure S1A) compared with TLR4**^+/+^** mice. While both strains swam similar distances (F_(*1,164)*_ = 2.10, *p* = 0.1550) (Figure S1B), TLR4^−/−^ mice exhibited significantly faster swim speed (F_(*1,164)*_ = 9.94, *p* = 0.0141) (Figure S1C), correlated with poorer path efficiency (the ratio of the shortest possible path length to actual path length; higher path efficiency pertains to better performance) during trials (F_(1,164)_ = 7.29, *p* = 0.0076) (Figure S1D). This is potentially due to their faster swim speed, which results in a greater area covered in a trial. To test the possible effect of swim speed on the performance of mice in this test, a correlation matrix including all measures with significant main effects (swim speed, latency to reach the hidden platform, mean distance from the platform) was performed. Results revealed that both latency to reach the hidden platform and mean distance from the hidden platform were correlated with swim speed (*r* = 0.57, *p*<0.0001 and *r* = 0.31, *p*<0.0001 respectively) (Figures S1E, F respectively). Because performance in the MWM test strongly depends on swim speed, the enhanced performance of TLR4^−/−^ mice compared with TLR4^+/+^ mice in the MWM task may have been the result of the higher swim speed of TLR4^−/−^ mice. To control for this possibility, an analysis of covariance (ANCOVA) was performed, in which swim speed was co-varied out of the latency to reach the hidden platform and mean distance from the hidden platform effects. The difference in mean distance from the hidden platform was still significant (*F*(1,215)  = 5.4, *p*<0.021) while the difference in latency was no significant (*F*(1,215)  = 0.336, *p*<0.563).

The higher swim speed of TLR4^−/−^ mice was also correlated with a significantly enhanced motor performance on the rotarod (F_(*1,72)*_ = 7.75, *p* = 0.008) (Figure S1G).

**Figure 2:**
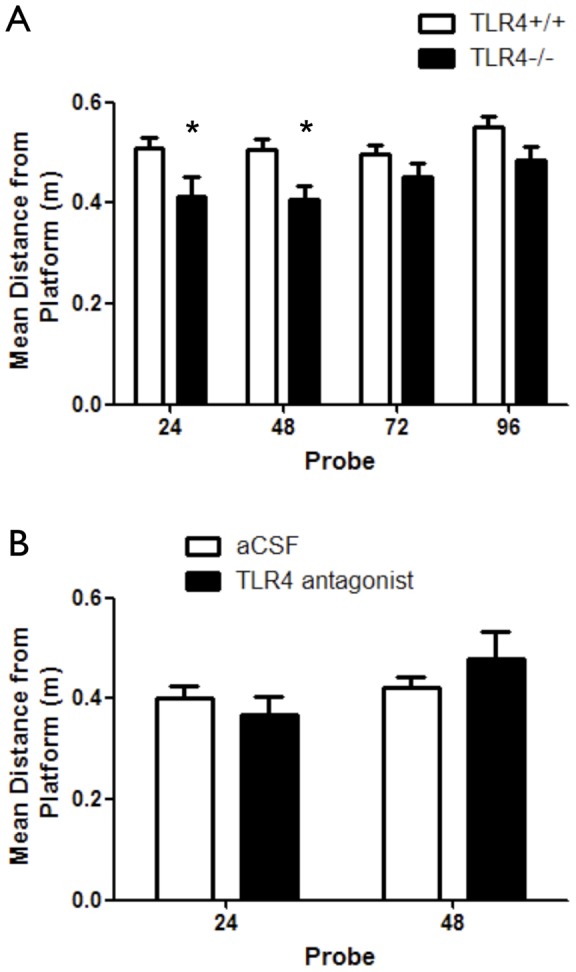
Developmental TLR4 deficiency, but not pharmacological TLR4 antagonism, enhances retention of spatial reference memory. (**A**) TLR4^−/−^ (n = 19) and TLR4^+/+^ (n = 24) mice were tested in probe trials at 24, 48, 72 and 96 hours following training for retention of spatial reference memory. Tests were done after all experimental groups exhibited loss of memory of the platform location. Mean distance from the platform was measured and used to indicate efficiency in locating the hidden platform. TLR4^−/−^ mice showed shorter mean distance from the platform at 24 and 48 hours after training compared with TLR4^+/+^ mice, indicating a more accurate swim toward the platform quadrant (**B**) Mice (C57BL/6) were implanted with an osmotic pump containing either aCSF (n = 10) or a TLR4 antagonist (n = 10). The pump was connected via tubing to a cannula, which was positioned to the lateral ventricle. Following training in the MWM task, mice were tested in probe trials at 24 and 48 hours following training for retention of spatial reference memory. Both experimental groups exhibited similar performance during probe trials, as measured by mean distance from the platform.

Spatial learning and memory tasks are comprised of several stages, including memory acquisition and memory extinction [16]. In probe trials to measure retention of the memory of the platform location, TLR4^−/−^ mice swam a shorter mean distance from the platform in probe trials conducted 24 and 48 hours after training compared with TLR4^+/+^ mice (*p*<0.05) (Figure 2A). These results could not be attributed to altered anxiety or exploratory levels as performance of these mice in the elevated plus maze task was similar (F_(*1,43)*_ = 0.28, *p* = 0.6) (Figure S1H), and no differences were observed in the open field arena (F_(*1,340)*_ = 0.03, *p* = 0.87) (Figure S1I).

Because TLR4 deficiency might affect development of the hippocampal circuits involved in cognition, we assessed the effect of pharmacological inhibition of TLR4 in mature mice to determine whether TLR4 mediates physiological hippocampal synaptic plasticity after the adult neural circuitry is established. To accomplish this, we infused either aCSF (vehicle control) or an antagonistic variant of LPS from *Rhodobacter Sphearoides* [17] into the lateral ventricles of adult TLR4^+/+^ mice (referred herein as TLR4 antagonist infused mice). This antagonistic LPS directly competes with hexa-acylated LPS for the same binding site on myeloid differentiation factor 2 (MD-2), as well as inhibits hexa-acylated endotoxin: MD-2 complexes function at TLR4 [13]. The infusion lasted 4 week, during which the first week was dedicated for animals' recovery and during the last 3 weeks the mice were tested. A concentration of 1 µg/µl was chosen for its ability to compete with saturated levels of exdotoxin or comparable ligand. Initially, the mice were tested in the reference memory variant of the MWM. The TLR4 antagonist did not affect mean distance from the platform (F_(1,68)_ = 0.08, *p* = 0.08) (Figure 1B). While swim distance or latency to reach the hidden platform were not different between the two groups (F_(1,68)_ = 0.01, *p* = 0.92 and F_(1,68)_ = 1, *p* = 0.3313 respectively) (Figures S2A and S2B respectively), swim speed was significantly lower in TLR4 antagonist infused mice (F_(1,68)_ = 9.73, *p* = 0.0063) (Figure S2C), which correlated with higher path efficiency during training (*p*<0.05) (Figure S2D). Retention of the memory of the platform location was not significantly different between the control and TLR4 antagonist-treated groups (Figure 2B). Despite no differences in spatial learning, anxiety levels were significantly different between the two treatment groups, as TLR4-antagonist infused mice showed significantly altered anxiety (more time spent in the open arms) as tested using the elevated plus maze (*p* = 0.0418) (Figure S2E) compared to aCSF infused mice. In addition, open field exploration analysis showed treatment differences in time spent in both periphery and center: TLR4 antagonist infused mice spent significantly more time in the periphery and less time in the center compared with aCSF infused mice (F_(1,180)_ = 7.29, *p* = 0.0076) (Figure S2F). During the 4 week period between cannulation and the end of behavioral tests, mice consistently gained weight and had overall good health (Figure S2G).

In a visible platform variant, aimed at assessing the impacts of motivation, visual abilities or motor function of the mice during performance in the MWM tasks described above, all four groups exhibited similar performance (TLR4^+/+^ vs. TLR4^−/−^, F_(1,42)_ = 0.82, *p* = 0.37, aCSF vs. TLR4 antagonist infused mice, F_(1,34)_ = 0.01, *p* = 0.9) (Figure S3A and B).

### Impact of TLR4 on hippocampus-dependent contextual fear learning and memory

**Figure 3:**
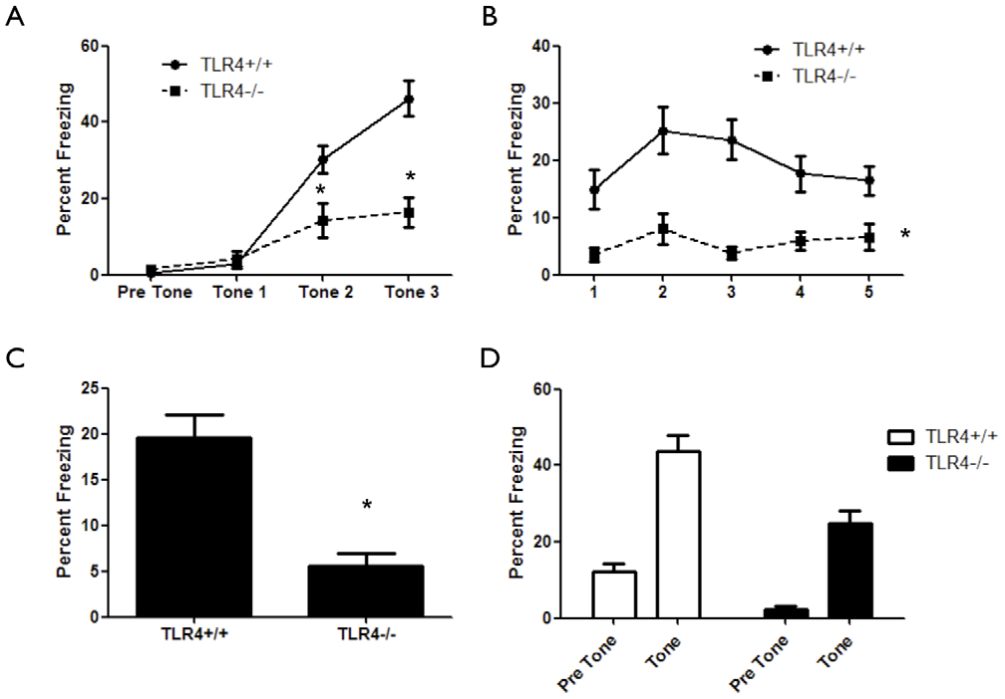
Developmental TLR4 deficiency impairs fear-learning and memory. (**A**) TLR4^−/−^ (n = 19) mice show impaired association curves in the fear-conditioning paradigm compared with TLR4^+/+^ (n = 24) mice. (**B**) TLR4^−/−^ mice exhibit significantly impaired hippocampus dependent contextual fear compared to TLR4^+/+^ mice, as measured by time freezing during 5 minutes of exposure to the original context. (**C**) Average freezing during contextual fear. (**D**) TLR4^−/−^ mice exhibit reduced freezing compared with TLR4^+/+^ mice in the presence of tone, indicating impaired fear response.

**Figure 4:**
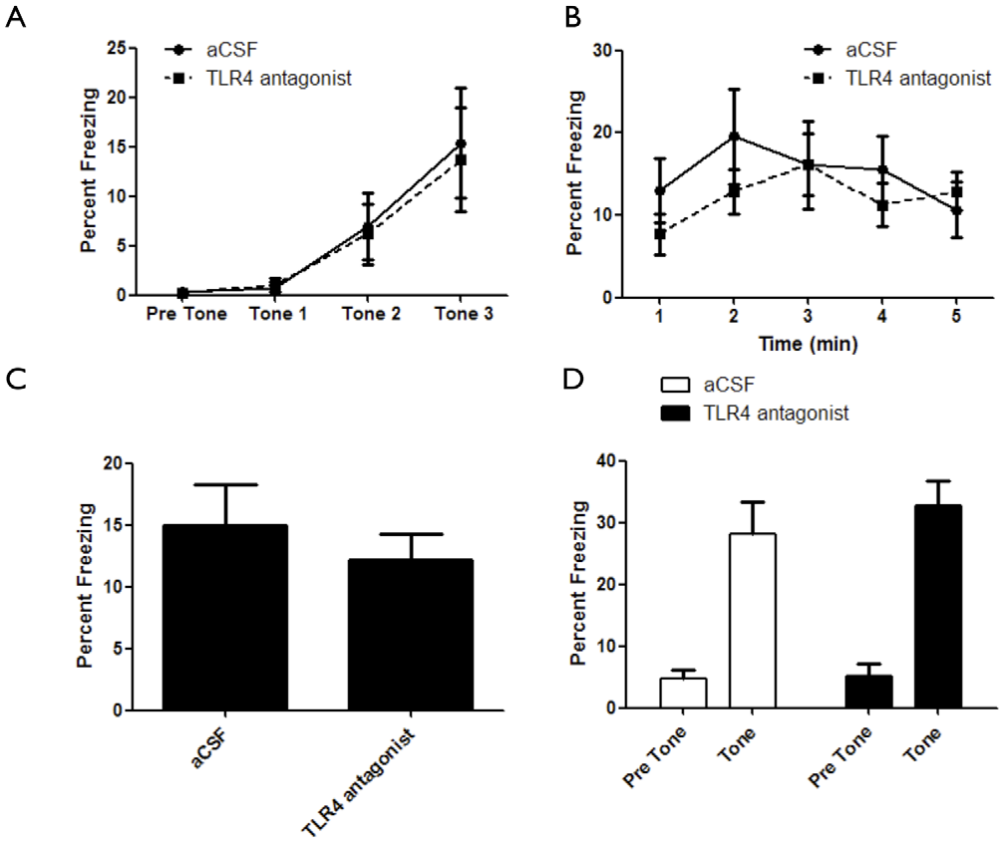
Pharmacological TLR4 inhibition does not affect fear-learning and memory. (**A**) aCSF infused mice (n = 10) show similar association curves in the fear-conditioning paradigm compared with TLR4 antagonist infused mice (n = 10). (**B**) aCSF infused mice (n = 10) show similar freezing levels in the fear-conditioning paradigm compared with TLR4 antagonist infused mice (n = 10). (**C**) Average freezing during contextual fear. (**D**) aCSF infused mice (n = 10) show similar freezing in the fear-conditioning paradigm compared with TLR4 antagonist infused mice (n = 10) in the presence of tone.

In addition to spatial learning, the hippocampus is involved in contextual fear learning [18]. Fear conditioning is a Pavlovian task shown to involve the hippocampal formation, the amygdala and the prefrontal cortex [19]. To test the hypothesis that hippocampal TLR4 has a role in contextual fear learning, TLR4^−/−^ mice were tested in the fear-conditioning paradigm. TLR4^−/−^ mice displayed an inability to associate the tone-shock pair compared with TLR4^+/+^ mice (F_(1,123)_ = 12.97, *p* = 0.0008) (Figure 3A). In addition, TLR4^−/−^ mice showed significantly lower freezing during contextual fear conditioning compared with TLR4^+/+^ mice (F_(1,164)_ = 20.41, *p*<0.0001) (Figure 3B, C). Similarly, TLR4^−/−^ mice showed significantly lower freezing during cued fear conditioning compared with TLR4^+/+^ mice, which could be attributed to initial lower freezing behavior in TLR4^−/−^ mice (F_(1,74)_ = 23.27, *p*<0.0001) (Figure 3D). Interestingly, inhibition of TLR4 in adult mice using an antagonist had no effect on acquisition (F_(1,60)_ = 0.06, *p* = 0.98) (Figure 4A), contextual memory (F_(1,64_ = 0.52, *p* = 0.48) (Figure 4B, C) or cued memory (F_(1,34)_ = 0.51, *p* = 0.47) (Figure 4D) in the fear conditioning paradigm.

### CREB levels are upregulated in brains of TLR4^−/−^ mice

**Figure 5:**
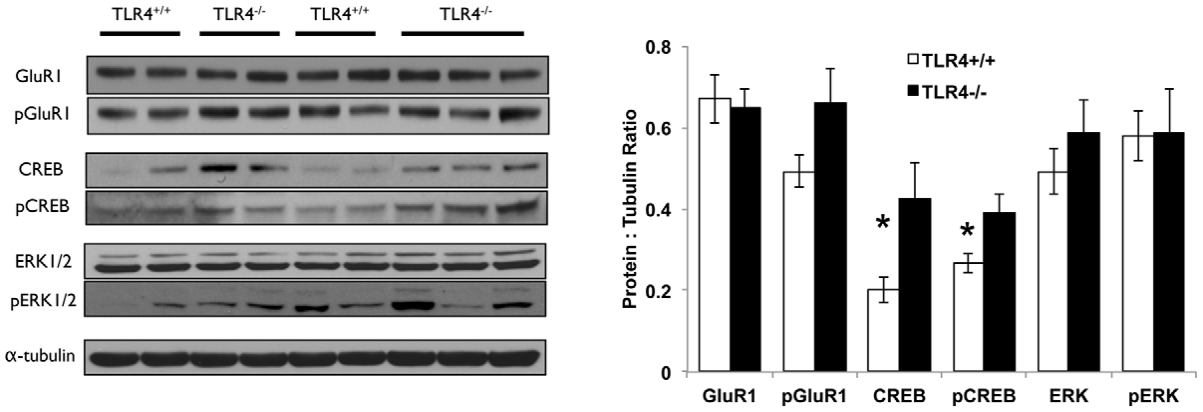
CREB and p-CREB are upregulated in the hippocampus of TLR4^−/−^ mice. Brains from TLR4^+/+^ (n = 8) and TLR4^−/−^ (n = 8) mice were dissected and hippocampi were removed. Tissues were then lysed, electrophoresed and immunoblotted against GluR1, CREB, ERK and their phosphorylated forms. Representative blots demonstrate that levels of CREB and pCREB were upregulated in TLR4^−/−^ mice compared to TLR4^+/+^ mice, whereas GluR1, ERK and their phosphorylated forms were not changed. * p<0.05.

To understand what could contribute to the altered cognitive ability of TLR4^−/−^ mice in spatial and contextual learning and memory paradigms, we compared cortices and hippocampi from TLR4^−/−^ and TLR4^+/+^ mice for expression of proteins previously found by us to be altered in response to TLR3 deficiency in the brain and that were correlated with cognitive behavioral changes [10]. In contrast to our observations in hippocampi of TLR3-deficient mice, the glutamate receptor (GluR1) – ERK pathway was not upregulated in the hippocampi of TLR4^−/−^ mice. Levels of CREB, the master-regulator of synaptic plasticity [20] and its phosphorylated form were elevated in hippocampi (Figure 5) but not cortices (Figure S4) of TLR4^−/−^ mice compared to TLR4^+/+^ mice.

## Discussion

TLR4, an innate immune receptor involved in the detection of gram-negative bacteria [3] and in tissue damage responses [21], has recently been suggested to play roles in CNS plasticity [6], [22]. Here we describe several roles for TLR4 in hippocampus-dependent learning and memory. We utilized mice devoid of TLR4, as well as adult mice infused with a TLR4 antagonist into the lateral ventricles in the brain, and a battery of behavioral tests to dissect the roles of TLR4 in hippocampus-dependent spatial as well as contextual learning and memory. To the best of our knowledge, the TLR4 antagonist we used throughout this study antagonizes TLR4 with a high affinity and specificity; however, it is possible that other, unknown signaling molecules, also bind this antagonist. Our findings suggest that TLR4 has a developmental role in shaping spatial and contextual learning and memory.

Developmental TLR4 deficiency, as exhibited in TLR4^−/−^ mice, conferred the strongest effect on hippocampus-dependent cognitive spatial and contextual learning and memory compared to pharmacological blockade of TLR4 in the adult brain. In a spatial reference memory task, TLR4^−/−^ mice showed enhanced performance compared to TLR4^+/+^ mice. On the other hand, pharmacological inhibition of TLR4 in adult mice did not alter spatial reference memory. Although TLR4^−/−^ mice swam faster in the reference memory variant of the MWM compared to TLR4^+/+^ mice, their shorter latency is attributed to better spatial navigation skills due to shorter mean distance from the platform and enhanced swimming strategy. The fact that developmental deficiency in TLR4 impacted spatial, contextual and motor learning is in line with previous studies reporting effects of TLR4 deficiency on the proliferation of NPC in the embryonic telencephalon as well as the postnatal and adult brain [6], [8]. In the latter studies, TLR4 deficiency promoted NPC proliferation, while TLR4 activation using LPS inhibited NPC proliferation. Further, TLR4 was shown to affect in-vivo neurogenesis in the dentate gyrus of adult mice by promoting differentiation of NPC into neurons. As TLR4 was implicated in adult dentate gyrus neurogenesis, and neurogenesis was implicated in cognitive learning and memory, we cannot rule out the possibility that the effects of TLR4 signaling on cognitive function are partially due to altered neurogenesis in the adult dentate gyrus.

The observed differences between the relative lack of effects on behavior of pharmacological inhibition of TLR4 in adult mice, compared to the effects of developmental TLR4 deficiency strongly suggests a developmental role for TLR4 in shaping the cellular and molecular substrates of learning and memory. TLR4 is expressed in NPC and neurons during development [6], [10], and could therefore affect the formation of neural circuits involved in cognition.

Interestingly, TLR4 inhibition in adult mice, but not developmental TLR4 deficiency, resulted in altered anxiety levels, as evident in performance in the open field arena and elevated plus maze task, suggesting that physiological TLR4 signaling may also be involved in anxiety/exploratory behaviors. Possible explanations for the latter results include; (1) chronic infusion of the TLR4 antagonist into the lateral ventricles results in diffusion of the antagonist to regions other than the hippocampus. (2) Anxiety is mediated by several brain structures such as the amygdala, cerebral cortex and locus coeruleus, as well as the hippocampus. When applying a TLR4 antagonist, the activation levels of TLR4 are altered, while in mice with developmental deficiency for TLR4, TLR4 is completely absent from the tissue. Moreover, while our results indicate that antagonizing TLR4 causes mice to spend a higher amount of time in the periphery of the open field arena, and decreases the amount of time they spend in the closed arms of the elevated plus maze, opposite effects are observed when TLR4 is activated using LPS in a rat model [23]; The rats spend a higher amount of time in the closed arms of the elevated plus maze and a higher amount of time in the center of the open field arena. This suggests that TLR4 is important for regulating normal anxiety responses.

It is possible that during embryonic development, different brain sub-regions important for fear conditioning are affected by developmental TLR4 deficiency, resulting in impaired fear learning in TLR4-deficient mice. The fact that only developmental TLR4 deficiency caused impairment in the fear-related learning and memory could also suggest that other brain sub-regions, such as the amygdala or the prefrontal cortex involve physiological rather than developmental effects by TLR4, which raises the need to study the role of TLR4 in these sub-regions as well.

Similarly, as motor function, tested using the rotarod task, was altered in TLR4^−/−^ mice but not mice with TLR4 antagonist infusion, brain regions important for coordination may develop differently under developmental TLR4 deficiency but are not affected by physiological TLR4 signaling. It remains to be studied whether TLR4^−/−^ mice exhibit structural anomalies in brain regions important for coordination such as the cerebellum.

Elevated CREB levels, correlated with the behavioral phenotype exhibited by TLR4^−/−^ mice, but were not correlated with several candidate pathways for CREB activation including ERK and GluR1. The molecular cascade from TLR4 to CREB therefore remains to be established. We hypothesize that increased CREB levels in TLR4^−/−^ mice have a functional role during development on synapse formation and brain structure. Further studies on the role of TLR4 on brain development during embryogenesis are needed to address the functional role of increased CREB activity in TLR4^−/−^ mice.

The data presented here expand our view of the role of TLR4 in learning and memory, and anxiety behaviors. However, further work will be required to understand the cellular and molecular mechanisms by which TLR4 signaling influences the formation and plasticity of neuronal circuits. It is unclear whether TLR4-dependent neurogenesis in the DG is responsible for the effects of TLR4 deficiency on spatial reference memory, and/or contextual fear learning and memory. As the most pronounced effects were conferred by developmentally TLR4 deficiency (improved motor ability (higher swimming speed and rotarod), improved spatial reference, impaired fear-learning), further examination of the roles of TLR4 in different brain regions during and following embryonic development is necessary.

It is interesting to compare the behavioral phenotype exhibited by mice lacking TLR3 to that of TLR4-deficient mice. Despite the structural and immunological functional similarities between the two receptors, the functional behavioral differences between the two receptors are striking. In contrast to TLR3^−/−^ mice, TLR4^−/−^ mice have enhanced acquisition of spatial reference memory. However, in contrast to TLR4^−/−^ mice, TLR3^−/−^ mice have slower spatial memory extinction. In fear-learning paradigms, TLR3^−/−^ mice show no impairment in memory acquisition, but show enhanced hippocampal and impaired amygdala component, while TLR4^−/−^ mice show impaired memory acquisition, impaired hippocampal and intact amygdala components. Finally, In contrast to TLR4 deficiency, TLR3-deficient mice exhibit impaired anxiety responses. These cognitive and anxiety differences are marked, and suggest inherent and critical roles for TLR-related genes during embryonic brain development. It will be interesting to see whether other members of this family of innate-immune receptors play roles in developmental neuroplasticity, learning and memory, and other behaviors.

## Supporting Information

Figure S1 **TLR4^−/−^ mice exhibit enhanced spatial reference memory, motor function and normal anxiety-like behavior.** TLR4^+/+^ (n = 24) and TLR4^−/−^ (n = 19) mice were trained for 5 days in the MWM with 4 trials per day. (**A**) Latency to reach the platform was significantly lower in TLR4^−/−^ mice compared with TLR4^+/+^ mice, (**B**) swimming distance was not different between the experimental groups and (**C**) mean swimming speed was significantly higher in TLR4^−/−^ mice compared with TLR4^+/+^ mice (**D**) Path efficiency was significantly lower in TLR4^−/−^ mice compared with TLR4^+/+^ mice (**E**) Pearson's correlation between swim speed and latency to reach the platform (**F**) Pearson's correlation between swim speed and mean distance from the platform (**G**) TLR4^+/+^ (n = 24) and TLR4**^−/−^** (n = 19) mice were tested in a Rota-Rod apparatus (Med-associates, St. Albans, VT, USA). Rota-rod acceleration was set to 4–40 revolutions per minute (RPM). Mice were placed on the Rota-rod for 3 trials of 5 minutes each with 15 minutes rest between trials. Time spent on the rod was measured. TLR4^−/−^ mice showed superior performance in this task compared with TLR4^+/+^ mice (**H**) TLR4^+/+^ (n = 24) and TLR4**^−/−^** (n = 19) mice were tested in an elevated plus maze. Mice were placed in the maze for 5 minutes, and time spent in the open and closed arms was measured. No difference was observed between the experimental groups (**I**) TLR4^+/+^ (n = 24) and TLR4**^−/−^** (n = 19) mice were tested in an open field arena. Mice were place in the center of the arena for 15 minutes, and time spent in the center versus the periphery of the arena was measured. No difference was observed between the experimental groups.
(TIF)

Figure S2 **CNS TLR4 inhibition affects anxiety but not spatial reference memory.** Mice implanted with osmotic pumps that infuse either aCSF (n = 10) or TLR4 antagonist (n = 10) were trained for 5 days in the MWM with 4 trials per day. (**A**) Latency to reach the hidden platform was not significantly different between the experimental groups, (**B**) Swim distance was not significantly different between the experimental groups, while (**C**) Swim speed was lower in TLR4 antagonist infused mice. (**D**) Path efficiency was significantly higher in TLR4-antagonist infused mice compared with aCSF infused mice at 48 hours after training (**E**) Mice implanted with osmotic pumps that infuse either aCSF (n = 10) or TLR4 antagonist (n = 10) were tested in an elevated plus maze. Mice were place in the maze for 5 minutes, and time spent in the open and closed arms was measured. TLR4 antagonist infused mice show altered anxiety response compared with aCSF infused mice (**F**) Mice implanted with osmotic pumps that infuse either aCSF (n = 10) or TLR4 antagonist (n = 10) were tested in an open field arena. Mice were place in the center of the arena for 15 minutes, and time spent in the center versus the periphery of the arena was measured. TLR4 antagonist infused mice show altered anxiety response compared with aCSF infused mice (**G**) Weight of mice following surgical procedure and during the 4 weeks in which the pumps infused aCSF or TLR4 antagonist into their lateral ventricles. Both experimental groups accumulated similar weights during the month of behavioral tasks.
(TIF)

Figure S3 **TLR4 expression had no impact on motivation, vision or motor function in spatial tasks.** Mice of the following interventions were placed in the water maze while the platform was visible, and were allowed to reach the platform during 4 consecutive attempts for 3 days. (**A**) TLR4^+/+^ (n = 24) and TLR4^−/−^ (n = 19) mice (**B**) Mice implanted with osmotic pumps that infuse either aCSF or TLR4 antagonist (n = 10 per group). No difference was observed between the different experimental groups.
(TIF)

Figure S4 **CREB, GluR1 and ERK are not altered in their expression levels in the cerebral cortex of TLR4^−/−^ mice compared with TLR4^+/+^ mice.** Brains from TLR4^+/+^ (n = 8) and TLR4^−/−^ (n = 8) mice were dissected and cortices were removed. Tissues were then lysed, electrophoresed and immunoblotted against GluR1, CREB, ERK and their phosphorylated forms. Representative blots are presented for the cerebral cortex. No significant difference was observed between CREB, GluR1, ERK and their phosphorylated forms between TLR4^−/−^ and TLR4^+/+^ mice. * p<0.05.
(TIF)
