# Supplementary figures and images for: Evidence for a Developmental Role for TLR4 in Learning and Memory
Source: PLoS One. 2012 Oct 11;7(10):e47522. doi: 10.1371/journal.pone.0047522 (PMC3469493; doi:10.1371/journal.pone.0047522)

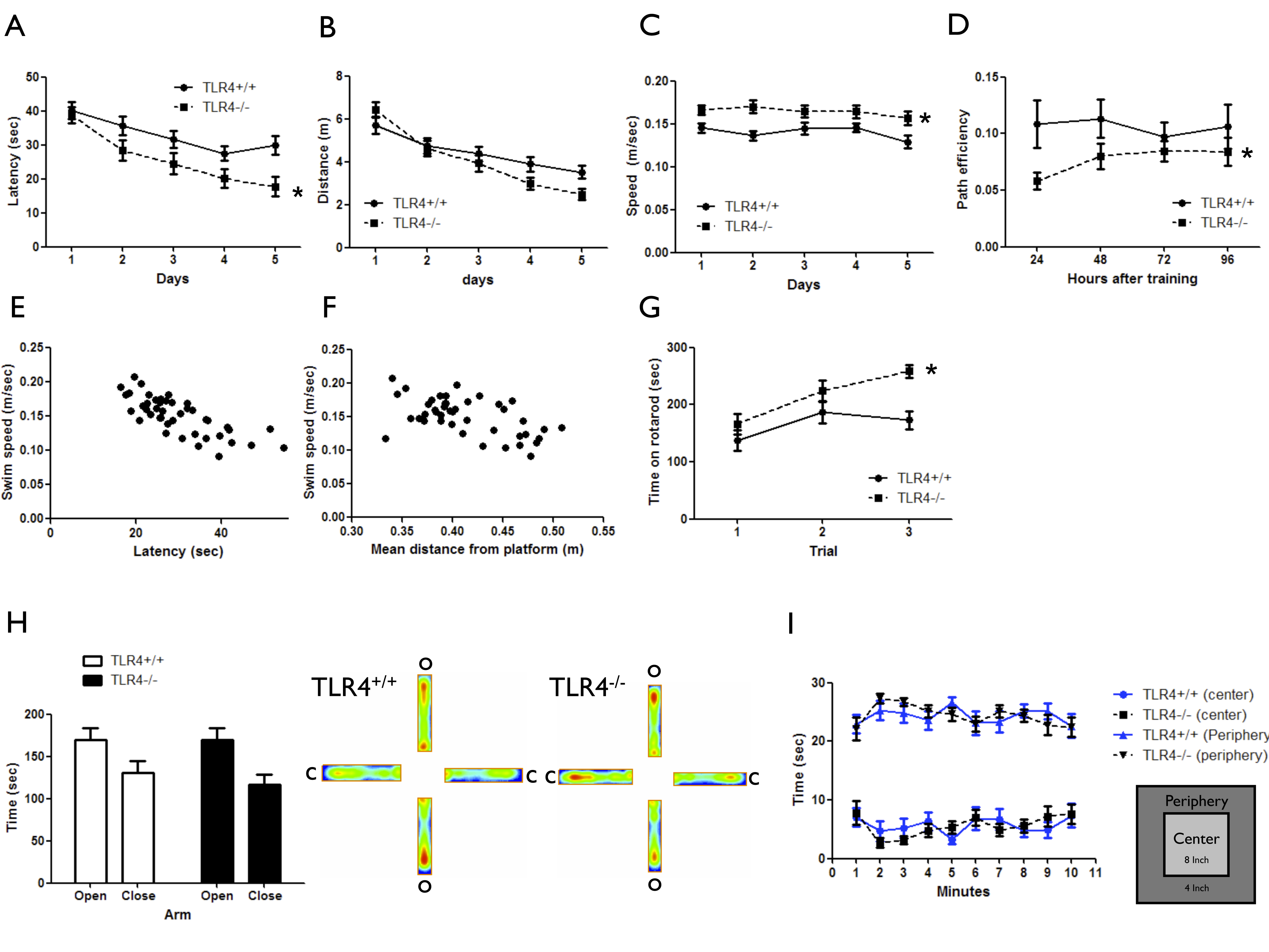

Supplement: Figure S1 — TLR4−/− mice exhibit enhanced spatial reference memory, motor function and normal anxiety-like behavior. TLR4+/+ (n = 24) and TLR4−/− (n = 19) mice were trained for 5 days in the MWM with 4 trials per day. (A) Latency to reach the platform was significantly lower in TLR4−/− mice compared with TLR4+/+ mice, (B) swimming distance was not different between the experimental groups and (C) mean swimming speed was significantly higher in TLR4−/− mice compared with TLR4+/+ mice (D) Path efficiency was significantly lower in TLR4−/− mice compared with TLR4+/+ mice (E) Pearson's correlation between swim speed and latency to reach the platform (F) Pearson's correlation between swim speed and mean distance from the platform (G) TLR4+/+ (n = 24) and TLR4−/− (n = 19) mice were tested in a Rota-Rod apparatus (Med-associates, St. Albans, VT, USA). Rota-rod acceleration was set to 4–40 revolutions per minute (RPM). Mice were placed on the Rota-rod for 3 trials of 5 minutes each with 15 minutes rest between trials. Time spent on the rod was measured. TLR4−/− mice showed superior performance in this task compared with TLR4+/+ mice (H) TLR4+/+ (n = 24) and TLR4−/− (n = 19) mice were tested in an elevated plus maze. Mice were placed in the maze for 5 minutes, and time spent in the open and closed arms was measured. No difference was observed between the experimental groups (I) TLR4+/+ (n = 24) and TLR4−/− (n = 19) mice were tested in an open field arena. Mice were place in the center of the arena for 15 minutes, and time spent in the center versus the periphery of the arena was measured. No difference was observed between the experimental groups. (TIF) [file pone.0047522.s001.tif]

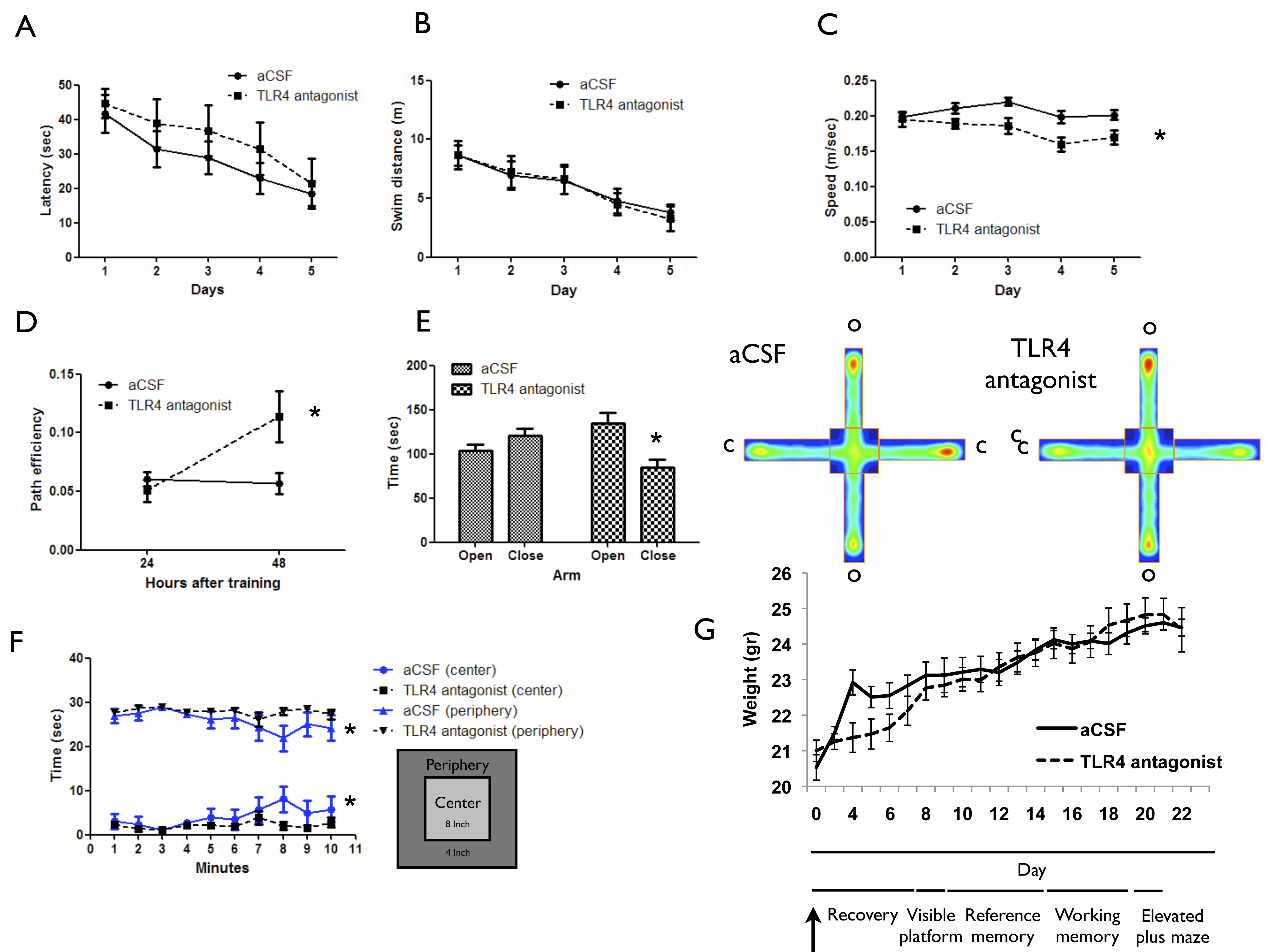

Supplement: Figure S2 — CNS TLR4 inhibition affects anxiety but not spatial reference memory. Mice implanted with osmotic pumps that infuse either aCSF (n = 10) or TLR4 antagonist (n = 10) were trained for 5 days in the MWM with 4 trials per day. (A) Latency to reach the hidden platform was not significantly different between the experimental groups, (B) Swim distance was not significantly different between the experimental groups, while (C) Swim speed was lower in TLR4 antagonist infused mice. (D) Path efficiency was significantly higher in TLR4-antagonist infused mice compared with aCSF infused mice at 48 hours after training (E) Mice implanted with osmotic pumps that infuse either aCSF (n = 10) or TLR4 antagonist (n = 10) were tested in an elevated plus maze. Mice were place in the maze for 5 minutes, and time spent in the open and closed arms was measured. TLR4 antagonist infused mice show altered anxiety response compared with aCSF infused mice (F) Mice implanted with osmotic pumps that infuse either aCSF (n = 10) or TLR4 antagonist (n = 10) were tested in an open field arena. Mice were place in the center of the arena for 15 minutes, and time spent in the center versus the periphery of the arena was measured. TLR4 antagonist infused mice show altered anxiety response compared with aCSF infused mice (G) Weight of mice following surgical procedure and during the 4 weeks in which the pumps infused aCSF or TLR4 antagonist into their lateral ventricles. Both experimental groups accumulated similar weights during the month of behavioral tasks. (TIF) [file pone.0047522.s002.tif]

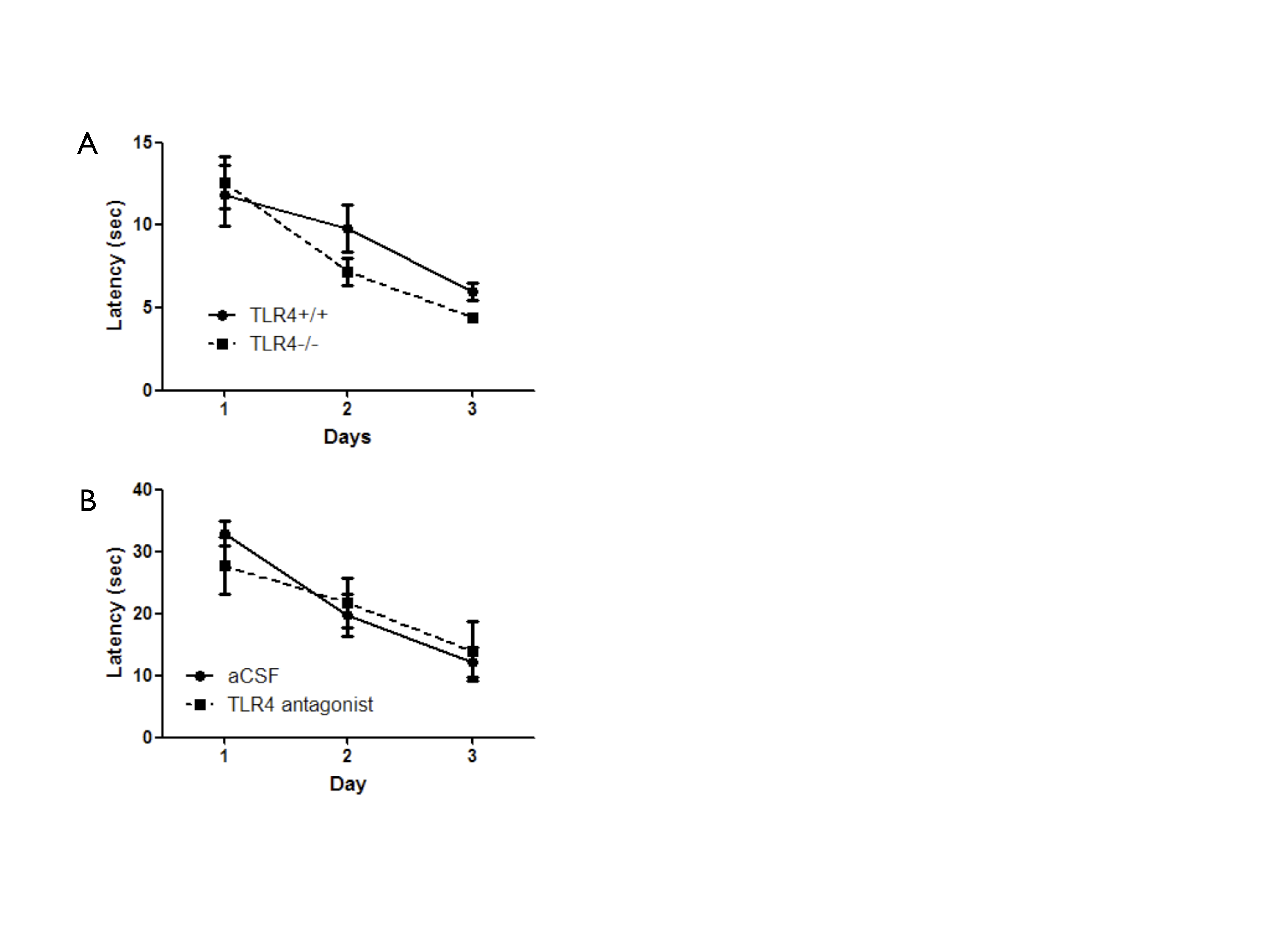

Supplement: Figure S3 — TLR4 expression had no impact on motivation, vision or motor function in spatial tasks. Mice of the following interventions were placed in the water maze while the platform was visible, and were allowed to reach the platform during 4 consecutive attempts for 3 days. (A) TLR4+/+ (n = 24) and TLR4−/− (n = 19) mice (B) Mice implanted with osmotic pumps that infuse either aCSF or TLR4 antagonist (n = 10 per group). No difference was observed between the different experimental groups. (TIF) [file pone.0047522.s003.tif]

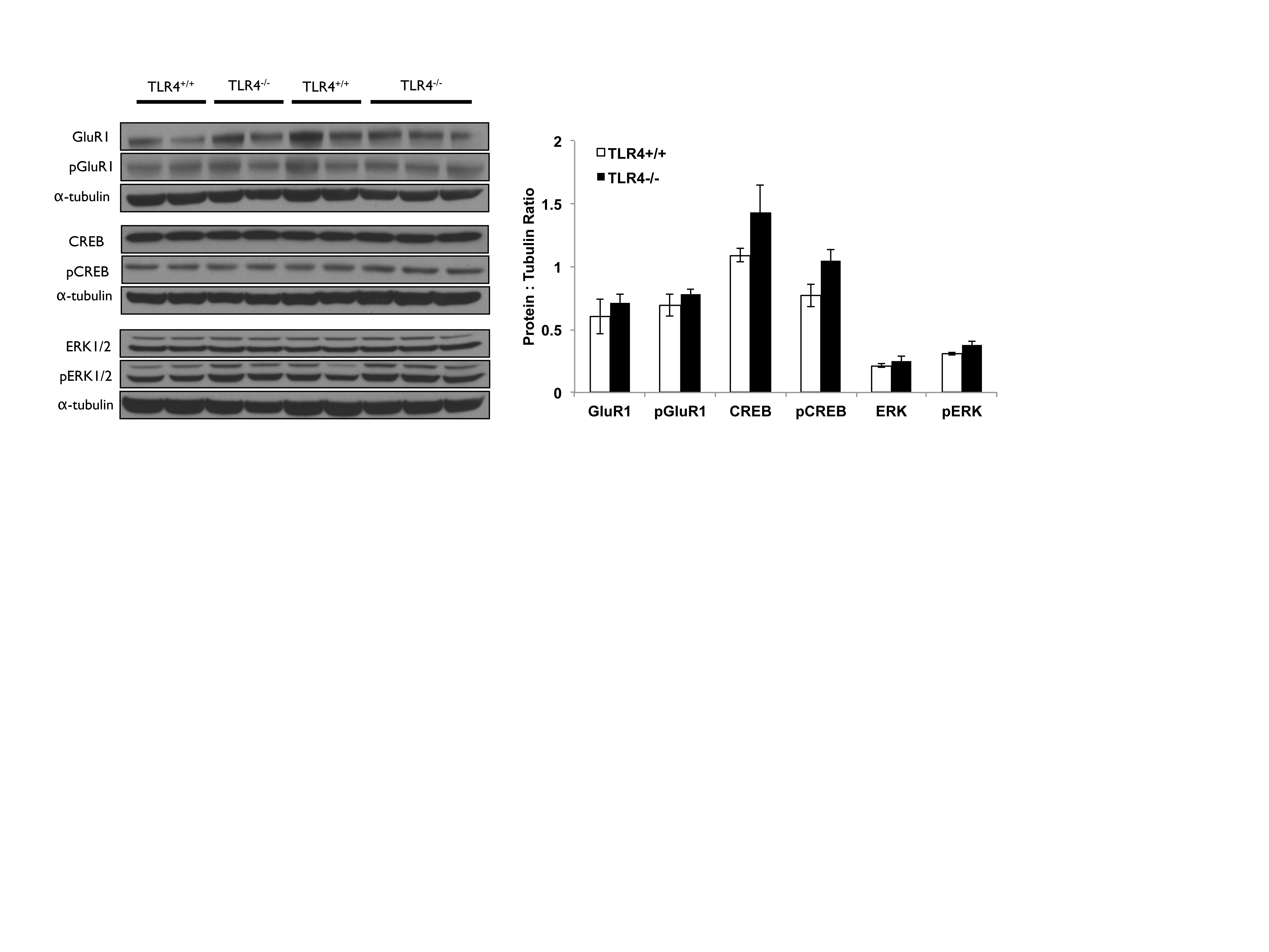

Supplement: Figure S4 — CREB, GluR1 and ERK are not altered in their expression levels in the cerebral cortex of TLR4−/− mice compared with TLR4+/+ mice. Brains from TLR4+/+ (n = 8) and TLR4−/− (n = 8) mice were dissected and cortices were removed. Tissues were then lysed, electrophoresed and immunoblotted against GluR1, CREB, ERK and their phosphorylated forms. Representative blots are presented for the cerebral cortex. No significant difference was observed between CREB, GluR1, ERK and their phosphorylated forms between TLR4−/− and TLR4+/+ mice. * p<0.05. (TIF) [file pone.0047522.s004.tif]
